# *In vivo* cranial bone strain and bite force in the agamid lizard *Uromastyx geyri*

**DOI:** 10.1242/jeb.096362

**Published:** 2014-06-01

**Authors:** Laura B. Porro, Callum F. Ross, Jose Iriarte-Diaz, James C. O'Reilly, Susan E. Evans, Michael J. Fagan

**Affiliations:** 1 Department of Organismal Biology and Anatomy, University of Chicago, 1027 East 57th Street, Chicago, IL 60637, USA; 2 School of Earth Sciences, University of Bristol, Wills Memorial Building, Queen's Road, Bristol BS8 1RJ, UK; 3 Research Department of Cell and Developmental Biology, University College London, Gower Street, London WCIE 6BT, UK; 4 School of Engineering, University of Hull, Cottingham Road, Hull HU6 7RX, UK

**Keywords:** Feeding, Skull, Biomechanics, Squamates

## Abstract

*In vivo* bone strain data are the most direct evidence of deformation and strain regimes in the vertebrate cranium during feeding and can provide important insights into skull morphology. Strain data have been collected during feeding across a wide range of mammals; in contrast, *in vivo* cranial bone strain data have been collected from few sauropsid taxa. Here we present bone strain data recorded from the jugal of the herbivorous agamid lizard *Uromastyx geyri* along with simultaneously recorded bite force. Principal and shear strain magnitudes in *Uromastyx geyri* were lower than cranial bone strains recorded in *Alligator mississippiensis*, but higher than those reported from herbivorous mammals. Our results suggest that variations in principal strain orientations in the facial skeleton are largely due to differences in feeding behavior and bite location, whereas food type has little impact on strain orientations. Furthermore, mean principal strain orientations differ between male and female *Uromastyx* during feeding, potentially because of sexual dimorphism in skull morphology.

## INTRODUCTION

*In vivo* bone strains provide direct evidence of strain regimes (*sensu* Ross et al., 2011) in skeletal structures during function. Data collected from the skull during feeding reveal strain patterns produced by internal and external loads; specifically, muscle forces and reaction forces at the jaw joints and teeth. Strain orientations are used to reveal the load transfer path and infer the deformation regime of the skull, while variations in strain magnitude highlight areas that are more or less subjected to deformation and thus adapted to resist feeding forces. Comparing strain orientations, magnitudes and distributions in the skulls of different taxa reveals aspects of cranial architecture adapted to particular diets, feeding behaviors or factors other than feeding.

*In vivo* bone strain data have been recorded from the crania of primates (Hylander and Johnson, 1997; Hylander et al., 1987; Ross, 2001; Ross and Hylander, 1996; Ross et al., 2011), sheep (Thomason et al., 2001), pigs (Herring and Teng, 2000) and hyraxes (Lieberman et al., 2004). These studies reveal significant variation in bone strain magnitudes across the skull during feeding, with higher strains in the facial skeleton and mandible than in the braincase or circumorbital region (Hylander et al., 1991a; Hylander et al., 1991b; Ravosa et al., 2000a; Ravosa et al., 2000b; Ross and Hylander, 1996; Ross and Metzger, 2004). Such highly strained areas of the skull are likely to be better optimized to resist loads generated during feeding, where optimality is defined as maximum strength for minimum material. In contrast, areas of the skull exhibiting low strain magnitudes during feeding instead function to protect the brain or eyes (Heesy, 2005; Hylander and Johnson, 1992; Hylander and Johnson, 1997; Ravosa et al., 2000a; Ross, 1995a), serve as areas for muscle attachment (Ross, 1995b) or provide a rigid framework to keep respiratory pathways open (Ross, 1995b; Ross, 2001; Ross and Metzger, 2004).

*In vivo* bone strain data have been recorded from the skulls of few non-mammalian taxa. Strain recorded from the cranium (Metzger et al., 2005) and lower jaw (Porro et al., 2013) of *Alligator mississippiensis* reveal heterogeneity in strain magnitudes in the skull. On average, maximum principal strain (ε_1_) magnitudes in the *A. mississippiensis* skull during feeding are high compared with those recorded in mammals: all gage sites on the *A. mississippiensis* cranium experienced ε_1_ strains over 1000 microstrain (με, which are equal to 1×10^−6^ inches inch^−1^ or mm mm^−1^) during at least one loading condition (Metzger et al., 2005), while the grand mean across all gage sites in the lower jaw was over 900 με (Porro et al., 2013). ε_1_ strains measured in the frontal bone of *Varanus exanthematicus* during feeding ranged from 100 to 600 με, although values as high as 2000 με were recorded (Smith and Hylander, 1985). In contrast, many areas of mammalian crania never experience strain magnitudes over 100 με. Results from these sauropsids suggest that their cranial morphology may be better optimized to resist feeding forces than mammalian crania, possibly because their relatively smaller brains are housed within the bony framework of the skull (Curtis et al., 2011; Curtis et al., 2013).

Lepidosaur skulls exhibit diverse feeding adaptations including clade- and diet-specific differences in skull and tooth morphology, and cranial kinetic potential (Herrel et al., 2001a; Herrel et al., 2001b; Herrel et al., 2004; Herrel et al., 2007; Metzger, 2002; Metzger and Herrel, 2005; Rieppel and Labhardt, 1979; Robinson, 1976; Schwenk, 2000; Stayton, 2005; Stayton, 2006). *Uromastyx* is a genus of agamid lizards found in northern Africa, the Middle East and south-central Asia. It is primarily herbivorous and, over the past three decades, has become a model for analysis of skull form and function. *Uromastyx* is characterized by specialized skull morphology and an acrodont dentition in which the teeth are fused to the jaw bones. Teeth are not replaced in adults, resulting in the development of extensive wear facets (Robinson, 1976; Throckmorton, 1979). Unlike most sauropsids, *Uromastyx* engages in cyclic intra-oral food processing (i.e. chewing), resulting in food being broken into smaller pieces. Chewing is distinct from mastication, a term reserved for mammals, as the latter involves transverse movements of the teeth during the power stroke and precise tooth–tooth occlusion (Throckmorton 1976; Throckmorton, 1980; Ross et al., 2007; Crompton, 1989). Chewing cycles in *Uromastyx* are longer, slower and less rhythmic than in mammals (Throckmorton, 1980; Ross et al., 2007), involve retraction of the jaw during closure (Throckmorton, 1976), and possible rotation of the lower jaw about its long axis as the teeth come into occlusion (Throckmorton, 1974; Throckmorton, 1980). The cranium of *Uromastyx* exhibits streptostyly (antero-posterior rotation of the quadrate against the squamosal) (Throckmorton, 1976; Herrel and De Vree, 1999). Previous *in vivo* experimental work on *Uromastyx* has included descriptions of lower jaw, tongue and streptostylic movements during feeding (Throckmorton, 1976; Throckmorton, 1980; Herrel and De Vree, 1999), electromyographic analysis of jaw and hyolingual muscle activity (Throckmorton, 1978; Throckmorton, 1980; Herrel and De Vree, 2009), and measurements of bite force (Herrel and De Vree, 2009).

Additionally, *Uromastyx* has been modeled using both multibody dynamics analysis (MDA), to simulate rigid-body motion under feeding loads, and finite element analysis (FEA), to understand the internal mechanical behavior of the cranium during feeding (Moazen et al., 2008a; Moazen et al., 2008b; Moazen et al., 2009a; Moazen et al., 2009b). Advantages of these methods compared with experimental techniques include measuring strain throughout the entire structure and predicting variables that are difficult or impossible to measure *in vivo* (such as joint reaction forces). However, model accuracy is dependent on input parameters used in their construction (Gröning et al., 2013). MDA modeling requires kinematic data to drive feeding simulations, while simultaneously recorded electromyographic and bite force data can be used to test model accuracy. Similarly, the accuracy of FEA is improved with detailed knowledge of muscle architecture and activity and bone material properties, while bone strains predicted by FEA can be validated against experimental strain data. Validation studies comparing predictions from finite element model (FEM) skulls with *in vivo* data have been largely confined to mammals (Verrue et al., 2001; Ross et al., 2005; Ross et al., 2011; Strait et al., 2005), with the cranium and lower jaw of *Alligator* representing the only non-mammalian FEMs for which *in vivo* validation studies have been carried out (Metzger et al., 2005; Porro et al., 2013). Until *Uromastyx* MDA and FEA results are validated against experimental data, it is unclear how well model predictions reflect reality.

In this paper, we present simultaneous bone strains and bite forces obtained during feeding in *Uromastyx geyri* Müller 1922; these data represent the first measurements of *in vivo* cranial bone strain obtained for an herbivorous sauropsid. The aims of this study are to: (1) document *in vivo* bone strains in the *U. geyri* cranium during feeding; (2) examine variations in strain magnitudes, principal strain orientations and ratios among individuals and with changes in bite location, food type and feeding behavior; (3) compare strain magnitudes in *U. geyri* with those obtained from other sauropsids and mammals; and (4) examine the relationship between bite force and strain magnitude.

## RESULTS

In some cases, gages failed during experiments or movement artifacts rendered data unusable. As a result, different amounts of data are available for each animal and for individual gage sites. The individual Uro1 yielded the largest data set, followed by Uro5 and Uro7.

### Observations of feeding behavior

Feeding sequences (defined as the series of behaviors during the ingestion of a single food item) were composed of individual cycles representing eight distinct feeding behaviors. Eighty-nine feeding sequences were recorded from the three subjects. The number of cycles per sequence ranged from two to 40 cycles, with a mean of 14 cycles per sequence; these results are similar to those reported by Throckmorton (Throckmorton, 1980) in *U. aegyptius* and Herrel and De Vree (Herrel and De Vree, 1999) in *U. acanthinurus*. Data were recorded during 39 transducer bites in Uro5. In total, data were collected for 1364 individual cycles.

A typical feeding sequence began with tongue flicks, characterized by low gape angles and rapid protrusion and retraction of the tongue; the tip of the tongue either contacted the food directly or the floor in front of the food [referred to as ‘tasting’ by Throckmorton (Throckmorton, 1980); specified as tongue touches by Herrel et al. (Herrel et al., 1998), in which kinematics and electromyography are also described]. Food was acquired during capture cycles: the animal approached the food, the jaws were opened slightly and the tongue protruded with its tip curled ventrally so that the dorsum of the tongue contacted the food (Throckmorton, 1980; Herrel and De Vree, 1999). The tongue, with the food adhered to it, was pulled back into the mouth as the jaws opened quickly and widely and the food was grasped in the anterior jaws. The animals then exhibited a variable number of manipulation cycles: these were characterized by the jaws opening rapidly to a wide gape, sharp lateral and ventral movements of the head as well as rolling of the head about its long axis, followed by rapid jaw closure. The purpose of these manipulation cycles is to shift the food from the front of the jaws to a posterior region of the tooth row more suitable for reduction. Manipulation cycles were followed by chewing*:* compared with manipulation cycles, chewing involved little or no movement of the head (which was usually held with the palate horizontal), low gape angles, and slower jaw opening and closure. Chewing resulted in the food being broken or folded into smaller pieces. The tongue was used to position food during both manipulation and chewing cycles. Chewing cycles were often interspersed with manipulation cycles as the animal finished reducing a portion of the food (especially larger leaves) and moved new portions to a more posterior position along the tooth row. Swallowing cycles were followed by licking cycles [referred to as ‘cleaning’ by Throckmorton (Throckmorton, 1980)], in which the animal opened the jaws slightly, the tongue protruded just beyond the anterior margins of the jaws and then retracted, and the jaws closed.

Two additional feeding behaviors were documented. Crushing occurred when the animals ate Mazuri pellets. The animal shifted the pellet to a posterior position of the tooth row (using manipulation cycles), then closed the jaws slowly and powerfully until the pellet failed suddenly, fracturing into several pieces within and outside of the mouth. In rare instances, the animal would brace the lower jaw against the ground during crushing, presumably to allow neck flexor muscles to contribute to the bite force. Crushing described in other agamids involves numerous cyclic movements (Herrel et al., 1997); *U. geyri* exhibited only one or two crushing cycles during a feeding sequence. Crushing cycles were followed immediately by chewing or swallowing cycles. Tearing resulted when the animal held a food item (usually a leaf) down with a foreleg while using movements of the head and neck to tear pieces from it. This behavior was rare (only five instances were recorded).

Food was always ingested at the front of the jaws and shifted to a posterior position for reduction. In some instances, particularly when feeding on Mazuri pellets, animals shifted food to one side of the head for processing; in these cases, working and balancing sides were clear. In most instances, the food item protruded from both sides of the mouth during reduction. Thus, bite location could be clearly assigned to only 32% of all cycles recorded.

### Principal strain magnitudes and ratios

**Fig. 1.**
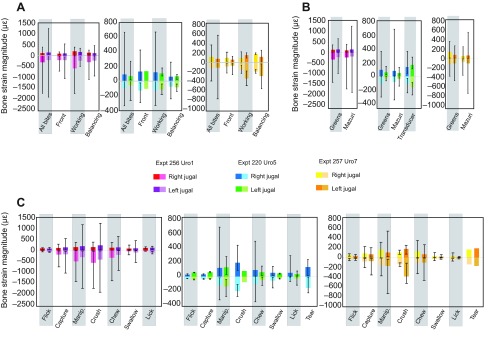
**Principal (ε_1_ and ε_2_) bone strain magnitudes (με) collected at right and left jugal sites in three *in vivo* experiments.** Data are sorted by bite point (A), food type (B) and feeding behavior (C); data for all recorded cycles are also shown (A). Bars indicate mean principal strain magnitudes; whiskers indicate peak strain magnitudes. Note that scales vary between experiments.

Mean and peak principal strain magnitudes were highest in Uro1, followed by Uro7 and Uro5 (supplementary material Tables S1–S3); the highest recorded principal strain (Uro1) was −1936 με. Working-side bites typically produced higher mean maximum (ε_1_) and minimum (ε_2_) principal strains than balancing-side or frontal bites (Fig. 1). Frontal bites produced the lowest mean strains in Uro1 and Uro7; balancing-side bites produced the lowest mean strains in Uro5. Feeding on greens produced higher mean ε_1_ and ε_2_ strains in all three individuals, although the highest peak principal strains occurred when feeding on Mazuri pellets. Transducer biting produced the highest mean principal strains in Uro5. In all three animals, tongue flicks, captures, swallows and licks produced low strains; the highest strains were generated during manipulation, crushing and chewing cycles.

Two-way mixed-model ANOVA (supplementary material Table S10) revealed significant effects of feeding behavior on ε_1_ and ε_2_ magnitudes at all analyzed gage sites. Individual differences account for significant variation in strain magnitude in the left jugal (48%) but not the right jugal (12%). Additionally, there were significant interaction effects between food type and behavior on ε_2_ magnitude (both sides) and ε_1_ magnitude (left jugal only). Food type typically did not significantly impact ε_1_ magnitude. *Post hoc* comparisons revealed that, at both gage sites, mean ε_1_ magnitudes elicited during manipulation and chewing were different from each other, and from those elicited during swallowing and licking; mean ε_1_ strain magnitudes during the latter two behaviors did not differ from each other. This was true for mean ε_2_ magnitudes, except that swallowing and licking differed from each other in the right gage. ANOVA testing for differences in principal strain magnitudes due to bite location revealed a significant effect of bite point at both gage sites (supplementary material Table S11).

The grand mean of maximum to minimum principal strain ratios (|ε_1_/ε_2_|) for all *in vivo* experiments was 1.76; within-gage means for all individuals was always >1, suggesting that tension is the predominant loading regime experienced by the *U. geyri* jugal during feeding (supplementary material Tables S1–S3). This is consistent with FEA of the *Uromastyx hardwickii* skull, which predicted that the ventral jugal is an area of high tensile strain during biting (Moazen et al., 2008a). The ε_1_/ε_2_ ratio was higher during frontal (Uro7, left gage of Uro5) or balancing-side bites (Uro1, right gage of Uro5) than during working-side bites; this is consistent with the expectation that anterior bites would load the jugal in tension while working-side bites would load the jugal in compression. Mean ε_1_/ε_2_ was >1 during transducer biting in Uro5, suggesting that tension was the predominant loading regime in the jugal; this is consistent with frontal bites placing the jugal in tension. Patterns in ε_1_/ε_2_ ratios were most consistent when data were sorted by feeding behaviors: tongue flicks, chews, licks and swallows nearly always produced ε_1_/ε_2_ ratios >1, indicating that the jugals experienced tension during these behaviors. Crushing always produced ε_1_/ε_2_ ratios <1, indicating that this behavior (which occurred in the posterior portion of the tooth row) compressed the jugals (supplementary material Tables S1–S3). ε_1_/ε_2_ ratios were highly variable during capture, manipulation and tearing cycles, most likely because of large and unpredictable head movements. Two-way ANOVA revealed significant effects of feeding behavior, as well as the interaction between food type and behavior, on ε_1_/ε_2_ ratios at both gage sites (supplementary material Table S10). ANOVA testing revealed significant differences in principal strain ratios due to bite location at both gage sites (supplementary material Table S11).

### Principal strain orientations

**Fig. 2.**
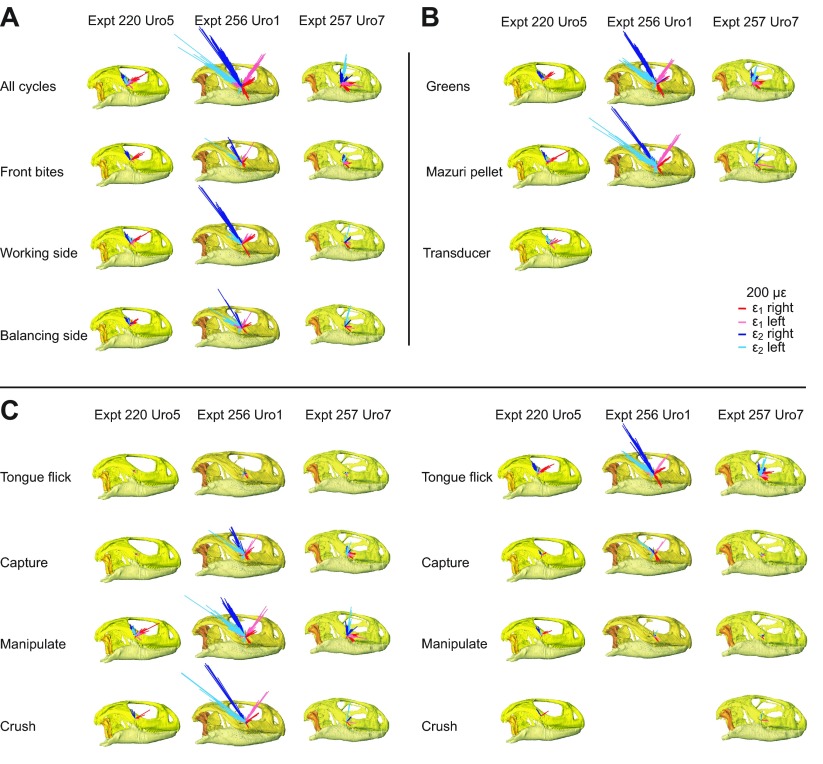
**Orientations of maximum (ε_1_) and minimum (ε_2_) principal strains at right and left gage sites (see inset) in three *Uromastyx geyri* individuals.** Left side strains are shown from the right for ease of comparison. Vector lengths correspond to principal strain magnitude, see scale bars; all vectors are shown to the same scale. Strain vectors are sorted by bite location (A), food type (B) and feeding behavior (C). Top row in A shows strain orientations for all recorded cycles.

Vector plots of strain orientation (Fig. 2) reveal two distinct strain regimes in the jugals of *U. geyri* during feeding, suggesting two separate loading regimes. For the three individuals, strain orientations were sorted by bite location, food type and feeding behavior to attempt to separate these loading regimes.

Mean ε_1_ orientation for all cycles in Uro1 was anteriorly directed in the right jugal and anterodorsally directed in the left jugal (supplementary material Table S2). On both sides, ε_1_ vectors during working-side bites were rotated counterclockwise compared with strains generated during frontal bites (supplementary material Table S2). The two strain regimes are most apparent when data are sorted by feeding behavior: for both right and left gages in Uro1, mean ε_1_ was generally directed anteroventrally during tongue flick, capture, lick and swallow cycles, and anterodorsally during manipulation, crushing and chewing (Fig. 2; supplementary material Table S2).

Mean vector length and concentration reveal that ε_1_ strain orientations were more concentrated during posterior bites than during frontal bites in Uro1 (supplementary material Table S4); additionally, ε_1_ strain orientations were most concentrated during crushing and licking cycles and most variable during capture cycles (supplementary material Table S6). Mardia–Watson–Wheeler tests demonstrate that ε_1_ orientations were significantly different with changes in bite location, food type and feeding behavior (supplementary material Tables S7–S9).

In Uro5, mean ε_1_ orientation for all cycles was anteriorly directed in the left jugal, and changes in ε_1_ orientations with bite location, food type and feeding behavior resembled trends observed in Uro1 (Fig. 2; supplementary material Table S1). In contrast, mean ε_1_ in the right jugal of Uro5 was anterodorsally directed and showed little variability with changes in bite location or food type. Mardia–Watson–Wheeler tests demonstrate that ε_1_ orientations were significantly different with changes in bite point (supplementary material Table S7) and food type (supplementary material Table S8) in the right jugal of Uro5.

Unlike Uro1 and Uro5, mean ε_1_ orientation in both jugals of Uro7 was anteroventrally directed (supplementary material Table S3). However, changes in ε_1_ orientation with bite location, food type and feeding behavior were asymmetric between the right and left jugals. In the right jugal, ε_1_ was rotated clockwise during posterior bites compared with frontal biting; the opposite pattern was observed in the left jugal (supplementary material Table S3). In the right jugal of Uro7, ε_1_ was rotated counterclockwise during tongue flick, capture and chewing cycles compared with other feeding behaviors; these behaviors caused clockwise rotation of ε_1_ in the left jugal (supplementary material Table S3).

As in Uro1, mean vector length and concentration reveal that ε_1_ strain orientations were less variable during posterior bites than during frontal bites on both sides of Uro7 (supplementary material Table S4). Right and left ε_1_ strain orientations were most concentrated during crushing in Uro7 (supplementary material Table S6). Mardia–Watson–Wheeler tests demonstrate that ε_1_ orientations were significantly different during feeding on different

foods (supplementary material Table S8) and between different feeding behaviors (supplementary material Table S9).

Two-way ANOVA confirms the significant effect of feeding behavior on ε_1_ orientation at both gage sites in Uro1 and Uro7 (supplementary material Table S12); additionally, it suggests that food type impacted strain orientations in Uro1 and the right jugal of Uro7. There were significant effects of bite location on ε_1_ orientation in Uro5 and Uro7 but not in Uro1 (supplementary material Table S13).

### Shear strain

Within-gage means of shear strain range from 124 to 1013 με, with the grand mean for all gage sites in all three individuals being 382 με. Peak shear strains recorded for each animal were 3195 με (Uro1), 981 με (Uro5) and 1661 με (Uro7). Two-way ANOVA (supplementary material Table S10) revealed significant effects of food type and feeding behavior on shear strain magnitude at right gage sites; at left gage sites, feeding behavior and the interaction between food type and behavior significantly impacted shear strain magnitude. ANOVA testing for differences in shear strain magnitudes due to bite location revealed significant effects at both gage sites (supplementary material Table S11).

### Bite force

**Fig. 3.**
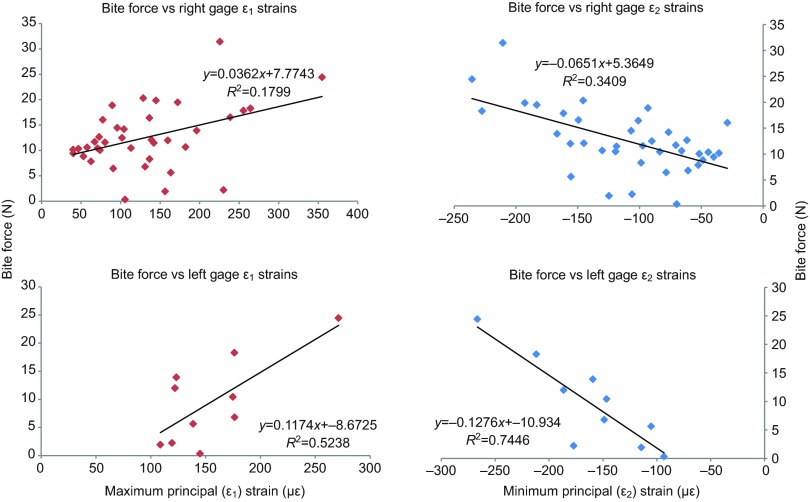
**Bite force plotted against simultaneously recorded maximum (left column) and minimum (right column) principal strain magnitudes for 39 transducer bites in Uro5 (Experiment 220).** Top row is strain data measured at the right gage; bottom row is data measured at the left gage. (Note that because of gage failure, a larger number of data points are available for the right gage than the left gage.)

The highest bite force produced by Uro5 was 31.4 N; mean bite force over 39 transducer bites was 12.5 N. These values are substantially lower than those reported in the similarly sized *U. acanthinurus* (59 N) or predicted in MDA models of *U. hardwickii* (51 N) (Herrel and DeVree, 2009; Moazen et al., 2008b). Most transducer bites occurred at the front of the jaws, although a small number of posterior bites were recorded; mean bite force recorded during posterior bites was higher (18.0 N) than during anterior bites (12.2 N). Bites were not prolonged; in some trials, several bites were delivered in quick succession. When bite force is plotted against simultaneously recorded maximum and minimum principal strains (Fig. 3), there is a positive correlation between bite force and both ε_1_ and ε_2_ strain magnitudes for left and right gage sites.

## DISCUSSION

We have presented new *in vivo* bone strain data from the cranium of *U. geyri*, the first herbivorous sauropsid and third species of lizard from which such data have been collected. Additionally, bone strain and bite force were recorded simultaneously for one *U. geyri* individual. These data were collected in order to: document *in vivo* bone strains during feeding in *U. geyri*; understand the impact of food type, bite location, feeding behavior and individual variation on bone strains; compare cranial strain magnitudes in *U. geyri* during feeding with those documented in other taxa; and document bite forces in *U. geyri*.

Some problems with the data set should be noted. First, only three *U. geyri* were of sufficient size for the collection of bone strain data (and only one of these was willing to bite on a force transducer), resulting in a small sample size. Second, owing to animal behavior and occasional gage failure, complete data sets are not available for

all experiments. Third, bone strains were collected from a single (bilateral) location – the jugal – as this was the only surface large enough to accommodate a gage that was also free of overlying muscle. Despite these drawbacks, our data set presents a substantial advance in our understanding of lizard cranial function during feeding.

### Variations in principal strain orientations in *U. geyri*

Mean principal strain orientations were remarkably consistent within individual *U. geyri* when all cycles were considered. ε_1_ orientation in the two males (Uro1 and Uro5) was anterodorsally directed in both the right and left jugal, suggesting compression of the postorbital bar along its long axis. In contrast, ε_1_ was anteroventrally directed in the female *U. geyri* (Uro7), suggesting anteroposteriorly aligned tension in the ventral jugal and shear in the postorbital bar. This overall intra-individual consistency in principal strain orientations cannot be attributed to local muscle forces as no muscles attach in this area; instead, it may reflect the overall deformation regime of the skull. Inter-individual variation is attributable to variation in gage location, skeletal morphology, bone material properties and feeding behavior.

Nonetheless, closer examination revealed two distinct strain regimes at nearly every gage site. Data were sorted to reveal the factors responsible for the underlying signal. In four of six gage sites, frontal bites generated ε_1_ strains rotated clockwise compared with posterior bites. This suggests that the postorbital bar is compressed parallel to its long axis during posterior bites, but not anterior bites. In their FEA of the *U. hardwickii* skull, Moazen et al. (Moazen et al., 2008a) found increased strain in the jugal (along with decreased strain in the skull roof) during posterior bites compared with anterior bites. They suggested that anterior bites induce compression in the bones of the snout and skull roof; in contrast, stress passes through the lateral aspect of the skull, including the jugal, during posterior bites. Our results are consistent with FEM predictions.

The clearest separation of the strain regimes occurred when data were sorted by behavior. ε_1_ strains were rotated clockwise during tongue flicks and capture cycles (four of six sites) and lick and swallow cycles (five of six sites) compared with manipulation, crushing and chewing cycles.

For Uro1 and Uro7, ε_1_ orientation was less variable during posterior bites than frontal bites, possibly because of the anterior jaws being used in more unpredictable behaviors. Circular–linear analyses reveal correlations between strain orientation and magnitude in Uro1 and Uro7 (supplementary material Tables S4– S6); such correlations are expected as there can only be one loading/strain regime during maximal contraction of the jaw muscles.

**Fig. 4.**
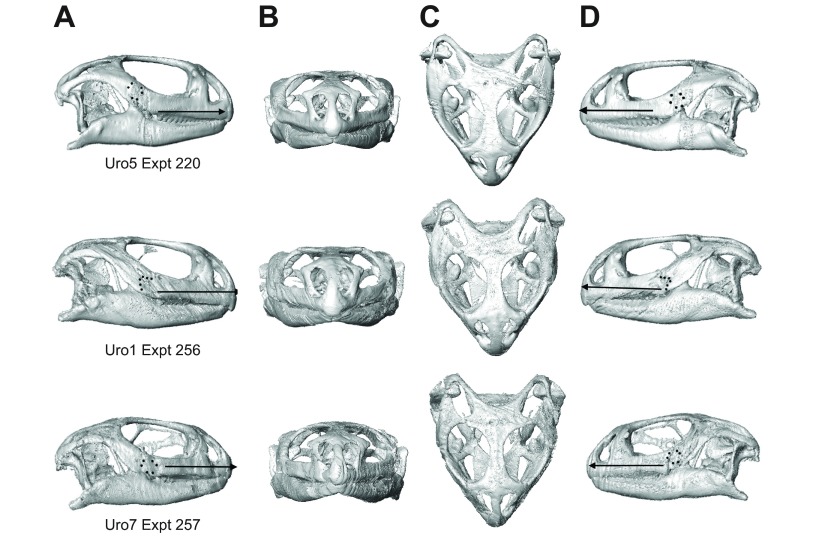
**3D renderings of the skulls of the three *Uromastyx geyri* individuals used to collect *in vivo* bone strain data.** Images were generated from CT scans (Gamma Medica Flex Triumph Imaging System, Department of Radiology, University of Chicago Medical Center) in Amira 5.4.2 (Visage Imaging, Berlin, Germany). Skulls are shown in right lateral (A), frontal (B), dorsal (C) and left lateral (D) views. Uro5 (top) and Uro1 (center) are male; Uro7 (bottom) is female. Lateral views illustrate the position and orientation of bone strain gages (black dots) and the reference axis (black arrow aligned with the anterior palate) that was used to standardize principal strain orientations across gage sites and experiments.

The difference in mean ε_1_ orientation between male and female *U. geyri* was consistent for both sides of the head. Comparison of gage locations using CT scans and radiographs rules out the possibility that this was due to subtle differences in gage position. It is possible that variations in bone material properties may be responsible; this suggestion awaits testing. When compared with the smaller male (Uro5), the female (Uro7) exhibits a relatively shorter head, more rounded skull table and more vertical postorbital bar (Fig. 4); these differences are more pronounced when Uro7 is compared with the large male (Uro1). Differences in bite force and diet have been attributed to sexual dimorphism in lizard skulls (Herrel et al., 2001a; Herrel et al., 2001b; Kaliontzopoulou et al., 2012;

Lappin et al., 2006), and it is not surprising that dimorphism would lead to differences in loading and strain regimes in males and females of the same species, although this study is the first to document such a difference. Because of the small sample size (two males and one female), further experiments are needed to confirm these results.

### Strain magnitudes in *U. geyri* and comparisons with other taxa

Strain magnitudes (Fig. 1) and results from ANOVAs suggest that inter-individual effects have the greatest impact on principal and shear strain magnitudes in the *U. geyri* cranium during feeding, followed by feeding behavior, bite location and food type.

Mean strain magnitudes across all three *U. geyri* were 102 με (ε_1_), −147 με (ε_2_) and 382 με (shear), with peak principal strains exceeding ±500 με in Uro5 and Uro7 and approaching −2000 με in Uro1. Peak shear strains approached or exceeded 1000 με in Uro5 and Uro7 and exceeded 3000 με in Uro1. Mean principal strains in the *Alligator* skull frequently approached 1000 με (Metzger et al., 2005; Porro et al., 2013). Thus, mean principal in the cranium of *U. geyri* are not as high as those reported in *A. mississippiensis*; however, the latter is a carnivorous taxon that captures and subdues live, active prey (often with violent shaking or rolling). In contrast, *Uromastyx* is an herbivore not accustomed to biting defensively (although male *Uromastyx* do fight vigorously). It is possible that during the course of our experiments we did not elicit behaviors that resulted in near-maximum cranial bone strains. Alternatively, it is possible that *Uromastyx* crania are normally subjected to lower bone strain magnitudes during feeding. The higher safety factor may serve to protect the cranium against fatigue failure associated with the repetitive loading associated with herbivory. The importance of repetitive loading is also reflected in the fact that *Uromastyx* jaw elevator muscles contain more slow fibers than insectivorous lizard species (Herrel et al., 1998).

Comparisons with mammals are complicated by differences in skull shape and muscle attachments. Mean principal strains recorded from the maxilla of sheep during feeding were typically less than ±100 με, with peak strains around ±300 με (Thomason et al., 2001).

Mean principal strains recorded from the maxilla of pigs during mastication ranged from ±100 to ±300 με, while strains from the zygomatic arch were higher (Herring et al., 2001). Principal strains from the zygomatic arch of the hyrax during feeding rarely exceeded ±300 με (Lieberman et al., 2004), but in individual primates they average greater than 900 με, and can reach over 2000 με (Hylander and Johnson, 1997). However, it should be noted that the zygomatic arch of mammals is the site of origin for the masseter, unlike the jugal of reptiles. Thus, it would appear that the cranium of *U. geyri* experiences strain magnitudes similar to, if not higher than, those of sampled herbivorous mammals.

### Strain variability due to food type

Feeding on greens produced higher mean principal strains whereas feeding on Mazuri pellets resulted in higher peak strains (Fig. 1). Furthermore, at five of six gage sites, mean ε_1_ orientations are rotated counterclockwise during feeding on greens compared with feeding on Mazuri pellets (supplementary material Tables S1–S3). Differences in feeding behavior can explain these patterns. Although the average total number of cycles in a feeding sequence did not differ with changes in food, animals used a larger number of manipulation cycles when eating greens. In contrast, ingestion of Mazuri pellets involved more licking cycles and only pellets elicited crushing. At four of six gage sites, crushing produced higher strains than manipulation; as a result, pellet feeding sequences exhibited higher peak strains. However, no more than two crushing cycles were used during a feeding sequence, and the large number of low-magnitude licking cycles resulted in lower mean strains. Feeding sequences on greens involved more high-magnitude manipulation cycles, resulting in higher mean strains. Manipulation cycles exhibited ε_1_ orientations rotated counterclockwise relative to licking cycles at five of six gage sites; again, the different number of manipulation and licking cycles employed while processing different foods explains the mean overall strain orientations of these feeding sequences.

Why do *Uromastyx* change their feeding behavior with different foods? Manipulation cycles are more frequent during feeding on greens because of the larger size of the food item – as a portion of the leaf is reduced, manipulation cycles are used to bring a ‘fresh’ portion to the posterior tooth row. Fracture of the pellet (into many small fragments) during crushing results in more licking cycles. Crushing is employed to break down pellets (but not leaves) because of differences in their material properties: pellets are substantially harder and more brittle than leaves. Thus, differences in food size and material properties elicit changes in feeding behavior, which in turn result in different strain regimes in the jugal. These findings bolster the suggestion by Ross et al. (Ross et al., 2012) that a better understanding of the relationship between variables (including food properties, behavior and strain patterns) is necessary to elucidate the link between diet and skull morphology.

### Concluding remarks

The results presented here document *in vivo* bone strain in an herbivorous sauropsid for the first time. In addition to bone strain and bite force, 3D kinematic data, videofluoroscopic recordings and electromyographic data of jaw elevator muscles were obtained during these experiments and from an additional three *U. geyri* individuals. These results will be presented elsewhere and will be used to develop, refine and validate FEMs and MDA models of this taxon. Furthermore, the experiments described here are part of a larger collaborative study to collect *in vivo* experimental data from a range of lizard taxa exhibiting diverse skull and dental morphology, diets and feeding strategies (Gröning et al., 2013). The ultimate aim of these experiments and modeling efforts will be to quantitatively link specific variations in skull anatomy to varying functional demands in lepidosaurs.

The data presented here suggest that feeding behavior had greater impact on cranial strain orientation and magnitude than food type, highlighting the importance of sampling a wide range of behaviors to appreciate the stress, strain and loading regimes experienced by vertebrate skulls.

## MATERIALS AND METHODS

### Animal husbandry

Bone strain data were collected from three adult Saharan *Uromastyx geyri* housed at the University of Chicago (IL, USA). Subjects included two males, Uro1 [231 g; head length (HL, measured from premaxilla to posterior tip of squamosal) 36 mm] and Uro5 (212 g; HL 35 mm), and one female, Uro7 (202 g; HL 34 mm). Animals were housed individually or in sexed pairs in large (152×60×60 cm) molded plastic cages (Showcase Cages, Lake Elsinore, CA, USA) in a temperature-controlled room set at 21–24°C on a 12 h:12 h light:dark cycle. A basking spot at higher temperature (up to 49°C) was available to the animals. Once daily, animals were provided with leafy greens (lettuce, endive, bok choy, mustard greens) and Mazuri brand tortoise pellets *ad libitum*. Animals were kept on a substrate of white millet seed that was occasionally ingested intentionally with no ill effects. All husbandry and experimental procedures were in accordance with federal regulations and approved Institutional Animal Care and Use Committee protocols.

### Data collection

Bone strain data were recorded during three separate experiments (Table 1) using either stacked rectangular rosette strain gages (Uro1) (FRA 1-11-1L; Texas Measurements, Inc., College Station, TX, USA) or stacked delta rosette strain gages (Uro5 and Uro7) (SA-06-030WY-120; Vishay Precision Group Micro-Measurements, Raleigh, NC, USA). The gages were wired, insulated and sterilized with hydrogen peroxide gas using methods previously described (Ross, 2001; Ross et al., 2011). The lateral surface of the postorbital bar (formed by the jugal), which is located ventral and posterior to the orbit (Fig. 4), provided the only bone surface large enough for gage placement that was free of overlying jaw muscles. For all three animals, gages were placed on the lower portion of the jugal on both sides of the head. Gage positions and orientations were recorded using photographs, CT scans and radiographs (Fig. 4).

Animals were food-deprived for 24 h prior to surgery and anesthetized using an intramuscular injection of ketamine (15 mg kg^−1^ body mass) and dexmedetomidine (100 μg kg^−1^ body mass). After anesthesia, <1 cm^2^ of skin overlying the jugal gage sites was removed, the periosteum elevated to expose the bone, the bone degreased with 100% chloroform, and the gage bonded to the surface of the bone using cyanoacrylate adhesive. To prevent movements of the lead wires causing strain in the gage, wires were glued to the skin for ~1 cm using the same adhesive, gathered together and shallowly sutured to loose skin on the back. Anesthesia was reversed with atipamezole (100 μg kg^−1^ body mass) and the animals were returned to temporary

**Table 1.**
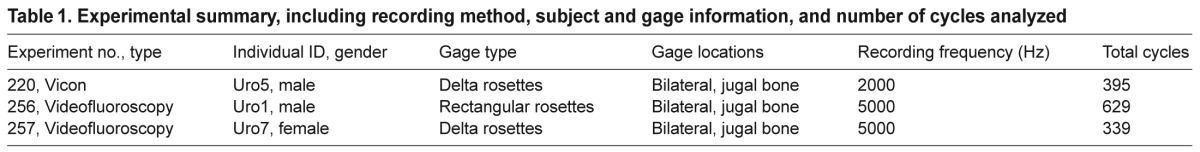
Experimental summary, including recording method, subject and gage information, and number of cycles analyzed

housing tanks to recover and for the duration of data collection. Data were collected twice daily (for up to 5 days) in sessions lasting no more than 2 h or until the animals stopped feeding.

The animals were fed in a clear Plexiglas tunnel (60×10×10 cm) while strain data were collected. For Uro5, experiments were recorded using a digital video camera; for Uro1 and Uro7, experiments were recorded using videofluoroscopy (General Electric OEC 9600 Series C-Arm, Fairfield, CT, USA). In both cases, strain data could be attributed to food type, bite location and feeding behavior. Voltage changes in the strain gages were conditioned and amplified on Vishay Micro-Measurements 2310A signal conditioning bridge-amplifiers while the animals fed on assorted greens (lettuce, endive and mustard greens) and Mazuri pellets. Food items were placed on the floor of the tunnel in front of the animals using tongs; occasionally, animals began eating while the item was held in the tongs. For Uro5, data were acquired at 2000 Hz and acquisition to PC was controlled by Vicon Nexus 1.6 Software (Vicon, Los Angeles, CA, USA). For Uro1 and Uro7, data were acquired at 5000 Hz through a National Instruments DAQ board run by MiDAS 2.0 Digital Video and Data Capture Software (Xcitex, Inc., Cambridge, MA, USA) and were saved to a PC for subsequent analysis.

*Uromastyx geyri* are not aggressive biters and bite force was obtained from only one individual (Uro5). *In vivo* bite forces were measured using a Kistler force transducer (Type 9203, range ±500 N; Kistler, Switzerland) connected to a Kistler charge amplifier (Type 5995) connected to an A-D system producing an output in voltage. Uro5 was manually restrained at the pectoral and pelvic girdles. The head was unrestrained during transducer bites; thus, deformation of the skull is attributed to muscle and bite reaction forces only.

### Bone strain data extraction

Strain gage outputs were filtered and processed in IGOR Pro 4.0.4 (WaveMetrics, Inc., Lake Oswego, OR, USA) using custom-written software and calibration files produced during the recording sessions. The strain data (strain being a dimensionless unit, ε, that represents change in length over original length, Δ*L*/*L*) were converted to με. The strain tracings (along with simultaneous video/videofluoroscopy and electromyograms) were examined to identify movement artifacts; these sequences were not included in the analysis. The magnitude of the maximum (ε_1_) and minimum (ε_2_) principal strains were calculated for every cycle recorded (Hibbeler, 2000); mean and peak principal strains recorded at each gage site in each experiment are recorded in Fig. 1 and supplementary material Tables S1–S3, sorted by bite location, food type and behavior. ε_1_ is the largest tensile (or occasionally least negative) strain and usually registers as a positive value; ε_2_ is the largest compressive (or occasionally least tensile) strain and usually registers as a negative value. The orientation of ε_1_ relative to the A-element of the strain gage was calculated for each cycle (the orientation of ε_2_ is orthogonal to that of ε_1_), as was the ratio of maximum to minimum principal strains |ε_1_/ε_2_|; values are presented in supplementary material Tables S1–S3. Shear strain (γ), which is equal to ε_1_–ε_2_, was also calculated for each cycle.

To facilitate comparisons between gage sites and experiments, strain orientations presented in all tables and figures (and used in statistical analyses) were calculated with the skull in right lateral view (thus, left side strains are seen from ‘below’). Strain orientations were calculated relative to the axes shown in Fig. 4; the reference axis (horizontal) is aligned with the palate in lateral view and is directed anteriorly whereas the vertical axis is perpendicular to this and points dorsally. By convention, positive values are rotated counterclockwise from the reference axis (vectors rotated clockwise from the axis are negative). Custom software within IGOR Pro 4.0 was used to convert strain orientations and magnitudes to vectors within polar coordinates. Vector plots (Fig. 2), in which the orientations and relative magnitudes of ε_1_ and ε_2_ during all recorded bites (as well as sorted by bite location, food type and behavior) are displayed, were created using Adobe Illustrator CS 5.1 (Adobe Systems Incorporated, San Jose, CA, USA).

### Bite force extraction

The force transducer used in Experiment 220 (Uro5) was calibrated by hanging weights of known mass from the transducer and recording the output voltage. The resulting linear regression (*y*=38.015*x*+0.2635; *R*=0.9964) was used to convert voltage to bite force. Bite force was plotted against simultaneously recorded maximum principal strains (ε_1_ and ε_2_) from both right and left gage sites (Fig. 3).

### Statistical analyses of bone strain data

To quantify the effects of various factors on strain magnitude and orientation, data from left and right gage sites were sorted by bite location (front, working side or balancing side), food type (greens, Mazuri pellets or force transducer) and feeding behavior. Missing data indicate that no strains were recorded for a particular bite location, food type or behavior.

Principal strain orientations are axial circular data in which an ε_1_ orientation of 0 deg is equal to 180 deg (and thus 90 deg is not a sensible mean). These data cannot be analyzed using traditional statistics. Quantitative analyses of *in vivo* principal strain orientations were performed in Oriana 3.13 (Kovach Computing Services, Anglesey, UK). In order to conduct these analyses, all angle data had to be converted to positive values (e.g. −30 deg was converted to 330 deg prior to analysis). Additionally, Oriana converts all axial data to values between 0 and 180 deg. Readers are urged to note these changes when comparing descriptive statistics from supplementary material Tables S1–S3 with circular statistics from supplementary material Tables S4–S6.

Descriptive circular statistics (supplementary material Tables S4–S6) were produced for ε_1_ orientations at each gage site, with data grouped by bite location, food type and behavior. Groups containing a single data point (see supplementary material Tables S1–S3) were excluded from statistical analyses. The statistics presented here include: the mean angle of the vectors (μ) relative to the reference axis describe above; the length of the mean vector (*r*) ranging from 0 to 1, which is a measure of angular dispersion with values closer to 1 indicating that individual observations are clustered more closely around the mean (length of mean vector is not the mean magnitude of ε_1_); the concentration (*k*), which measures the departure of the distribution from a uniform distribution (or perfect circle) and was calculated using published formulas (Fisher, 1993; Mardia and Jupp, 2000); the circular variance (*V*), which is calculated as *V*=1−*r*, and is equivalent to its linear counterpart; the circular standard deviation (*S*), calculated as *S*=[−2×ln(*r*)]^1/2^; the standard error of the mean; and the 95% and 99% confidence intervals derived from standard error. Additionally, Rayleigh's test of uniformity and Watson's *U*^2^-test were used to determine whether data are derived from a von Mises distribution (continuous probability distribution on a circle, not to be confused with von Mises stress). To determine whether ε_1_ strain orientations changed as strain magnitude increased, circular–linear correlation coefficients were calculated between ε_1_ orientation and magnitude (Zar, 1999) (supplementary material Tables S4–S6). To determine whether the distribution of ε_1_ angles differ significantly with changes in bite location, food type or feeding behavior, ε_1_ orientations recorded within each gage were compared using a non-parametric Mardia–Watson–Wheeler test or a parametric Watson–Williams *F*-test (supplementary material Tables S7–S9). (These tests determine whether two or more distributions are identical; significant differences between distributions will lead to a large *W* test statistic and low probability of distributions being identical.)

Mixed-model ANOVAs were used to investigate the effect of bite location, food type and feeding behavior on principal and shear strain magnitudes, and principal strain orientations in JMP 8 (SAS Institute, Cary, NC, USA) using the restricted maximum likelihood method, with individuals as random effects and food, behavior and their interaction as fixed effects (supplementary material Table S10). Because strain magnitude distribution was skewed, data were log-transformed to normalize them. Separate analyses were run for right and left gages; gage sites and behaviors with few data points were excluded. Because bite location was identified in only a third of all cycles, separate mixed-model ANOVAs were conducted for bite location with individuals as random effects (supplementary material Table S11). Tukey *post hoc* comparisons of differences in means were carried out. Significance was assessed at α=0.05. Angular data were analyzed using the CircStat (Berens, 2009) toolbox in MATLAB (MathWorks, Natick, MA, USA). Analyses were performed in the same groupings as above; however, because we cannot include individual variation as a random effect, we tested each individual independently. The effect of food type and behavior was analyzed using the Harrison–Kanji test (supplementary material Table S12). Depending on the concentration parameter, κ, two different statistics were used [χ^2^ and *F* for large κ; when κ is small, the interaction effect is not reported; see Harrison and Kanji (Harrison and Kanji, 1988)]. To test the effect biting side on strain orientation, we used the Watson–Williams test (supplementary material Table S13).

## Supplementary Material

Supplementary Material
