# Supplementary material for: In vivo cranial bone strain and bite force in the agamid lizard Uromastyx geyri
Source: J Exp Biol. 2014 Jun 1;217(11):1983–92. doi: 10.1242/jeb.096362 (PMC4059540; doi:10.1242/jeb.096362)
Supplement: Supplementary Material [file supp_217_11_1983__index.html]

Supplementary Material 

# *In vivo* cranial bone strain and bite force in the agamid lizard *Uromastyx geyri*

## JEB096362 Supplementary Material

**Files in this Data Supplement:**

- **Supplementary Material**
