# Supplementary material for: In vivo cranial bone strain and bite force in the agamid lizard Uromastyx geyri
Source: J Exp Biol. 2014 Jun 1;217(11):1983–92. doi: 10.1242/jeb.096362 (PMC4059540; doi:10.1242/jeb.096362)
Supplement: Supplementary Material [file supp_jeb.096362_JEB096362.pdf]

Table S1. Descriptive statistics for principal (ε_1 and ε_2) strain magnitude, $|(\varepsilon_1/\varepsilon_2)|$, and ε_1 orientation for Experiment 220 (*Uro5*).

Gage location	Bite side/ Food/ Behavior	n	ε_1			ε_2			$\varepsilon_1/\varepsilon_2$ ratio		ε_1 orientation	
			Mean	S.D.	Max	Mean	S.D.	Max	Mean	S.D.	Mean	S.D.
**Right jugal**	All	395	91	85	668	-95	77	-337	2.84	26.31	25	29
	Front	53	132	86	424	-129	72	-293	1.04	0.37	25	12
	WS	123	122	94	668	-129	77	-337	1.21	1.94	28	22
	BS	102	62	58	256	-75	64	-245	7.09	51.58	15	41
	Greens	221	93	77	353	-105	79	-293	4.06	35.13	25	31
	Mazuri	129	77	97	668	-77	77	-337	1.30	1.92	28	28
	Transducer	39	132	71	355	-109	55	-236	1.22	0.41	23	12
	Flick	4	25	9	39	-26	2	-28	0.96	0.32	53	6
	Capture	4	29	8	37	-31	5	-37	0.94	0.18	53	3
	Manipulation	128	118	94	668	-116	76	-313	1.04	0.48	29	26
	Crush	5	188	151	424	-194	66	-269	0.89	0.45	22	10
	Chew	175	87	79	477	-98	75	-337	4.56	39.41	22	31
	Swallow	11	51	42	137	-53	49	-152	1.29	0.55	43	24
	Lick	46	49	57	284	-50	68	-265	2.60	5.28	24	31
	Tear	4	130	90	231	-152	98	-218	1.07	0.49	26	10
**Left jugal**	All	67	67	54	271	-57	57	-266	1.75	1.48	1	29
	Front	1	139	NA	139	-105	NA	-105	1.32	NA	-38	NA
	WS	8	109	36	177	-113	50	-186	1.08	0.38	9	24
	BS	5	66	25	90	-85	42	-137	0.82	0.18	15	6
	Greens	15	56	33	144	-36	19	-62	2.64	2.68	17	34
	Mazuri	41	46	27	127	-40	37	-137	1.59	0.77	-4	23
	Transducer	11	162	50	271	-154	55	-266	1.15	0.54	-2	35

Flick	4	56	4	61	-38	7	-44	1.50	0.36	-9	5
Capture	3	60	9	69	-30	6	-37	2.02	0.10	-14	1
Manipulation	17	124	56	271	-130	59	-266	0.98	0.25	9	27
Crush	1	74	NA	74	-75	NA	-75	0.98	NA	6	NA
Chew	10	60	45	144	-45	45	-120	1.90	0.68	6	31
Swallow	4	46	5	49	-20	7	-29	2.47	0.73	-10	5
Lick	19	33	15	75	-28	24	-114	2.00	2.37	-2	30

Table S2. Descriptive statistics for principal (ε_1 and ε_2) strain magnitude, $|(\varepsilon_1/\varepsilon_2)|$, and ε_1 orientation for Experiment 256 (*Uro1*).

Gage location	Bite side/ Food/ Behavior	n	ε_1 Mean	ε_1 S.D.	ε_1 Max	ε_2 Mean	ε_2 S.D.	ε_2 Max	$\varepsilon_1/\varepsilon_2$ ratio Mean	$\varepsilon_1/\varepsilon_2$ ratio S.D.	ε_1 orientation Mean	ε_1 orientation S.D.
**Right jugal**	All cycles	543	123	72	390	-311	360	-1751	1.97	3.38	0	43
	Front	25	102	31	188	-192	215	-706	1.59	1.54	-4	46
	WS	62	192	109	390	-588	446	-1751	1.02	1.72	18	37
	BS	25	136	59	251	-297	314	-1099	2.88	6.04	17	34
	Greens	415	127	73	373	-335	361	-1444	1.84	3.24	1	42
	Mazuri	128	108	69	390	-233	344	-1751	2.39	3.80	-4	46
	Flick	9	94	29	127	-29	19	-69	5.27	4.36	-41	27
	Capture	48	109	46	283	-184	222	-753	1.87	1.85	-10	43
	Manipulation	136	148	78	365	-505	368	-1444	0.88	1.49	18	32
	Crush	23	155	110	390	-574	548	-1751	0.49	0.65	27	27
	Chew	214	121	76	373	-332	347	-1397	1.47	2.70	10	41
	Swallow	91	91	41	252	-37	29	-198	4.63	5.64	-41	33
	Lick	9	112	29	166	-31	7	-40	4.01	1.95	-54	3
**Left jugal**	All cycles	629	149	151	1261	-208	219	-1934	1.08	1.17	26	44
	Front	28	116	93	550	-191	200	-1049	0.78	0.37	32	44
	WS	25	166	75	333	-239	116	-496	0.75	0.39	52	18
	BS	65	116	76	463	-220	180	-944	0.78	0.63	30	49
	Greens	421	154	110	667	-217	189	-1010	0.97	0.66	36	41
	Mazuri	208	141	163	1261	-188	269	-1934	1.30	1.79	7	43
	Flick	13	85	22	133	-69	14	-97	1.24	0.27	-2	40
	Capture	56	137	93	550	-178	196	-1049	1.08	0.57	14	44

Manipulation	143	199	153	1190	-307	249	-1755	0.85	0.69	47	33
Crush	36	236	267	1261	-418	401	-1934	0.68	0.72	53	26
Chew	230	139	107	637	-198	177	-923	1.03	0.96	36	42
Swallow	120	105	72	446	-94	93	-557	1.56	2.05	-8	36
Lick	18	123	34	192	-99	31	-163	1.27	0.23	-28	3

Table S3. Descriptive statistics for principal (ε_1 and ε_2) strain magnitude, $|(\varepsilon_1/\varepsilon_2)|$, and ε_1 orientation for Experiment 257 (*Uro7*).

Gage location	Bite side/ Food/ Behavior	n	ε_1			ε_2			ε_1/ ε_2 ratio		ε_1 orientation	
			Mean	S.D.	Max	Mean	S.D.	Max	Mean	S.D.	Mean	S.D.
**Right jugal**	All	339	114	82	354	-104	77	-376	1.84	9.24	-29	34
	Front	30	95	52	211	-82	53	-213	1.39	0.58	-4	31
	WS	11	89	83	217	-144	63	-219	0.54	0.37	-46	13
	BS	5	176	13	189	-184	30	-220	0.97	0.14	-27	18
	Greens	275	130	78	354	-111	78	-376	2.14	10.24	-22	30
	Mazuri	64	44	54	242	-74	66	-358	0.57	0.30	-56	35
	Flick	10	51	17	80	-46	19	-66	1.26	0.54	-8	35
	Capture	51	86	62	226	-76	47	-209	1.19	0.61	-17	34
	Manipulation	103	165	74	314	-152	80	-376	1.24	0.78	-19	24
	Crush	4	105	37	154	-143	40	-183	0.77	0.28	-42	3
	Chew	108	130	80	354	-111	77	-333	1.72	3.20	-28	30
	Swallow	34	31	20	105	-30	16	-64	6.27	28.55	-59	39
	Lick	26	34	22	98	-53	16	-70	0.68	0.39	-55	37
	Tear	1	165	NA	165	-140	NA	140	1.18	NA	-42	NA
**Left jugal**	All	339	69	63	547	-105	113	-743	1.08	2.34	-26	35
	Front	30	59	29	125	-65	56	-245	1.14	0.61	-41	50
	WS	5	152	44	198	-324	112	-472	0.53	0.31	-13	6
	BS	11	113	83	252	-275	165	-534	0.37	0.15	-16	15
	Greens	275	72	50	265	-105	94	-472	1.04	1.83	-26	35
	Mazuri	64	56	101	547	-106	172	-743	1.21	3.84	-28	37

Flick	10	32	24	65	-28	15	-62	1.20	0.94	-33	56
Capture	51	63	42	200	-70	60	-359	1.07	0.58	-51	35
Manipulation	103	105	72	547	-170	122	-743	0.69	0.27	-15	20
Crush	4	175	79	252	-387	185	-534	0.48	0.10	-9	3
Chew	108	67	53	265	-105	103	-472	1.14	2.64	-24	33
Swallow	34	17	19	56	-15	11	-56	2.54	5.49	-37	51
Lick	26	9	5	20	-21	7	-30	0.50	0.34	-17	35
Tear	1	188	NA	188	-173	NA	-173	1.09	NA	-8	NA

Table S4. Descriptive circular statistics for *in vivo* bone strain orientations in the jugal of *Uromastyx*, sorted by bite location.

**Experiment and side**	**Exp 220 Right**			**Exp 220 Left**		**Exp 256 Right**		
**Bite point**	Front	WS	BS	WS	BS	Front	WS	BS
**Number of Observations**	53	123	102	8	5	25	62	25
**Mean Vector (μ)**	24.882°	31.5°	33.951°	12.012°	15.047°	32.143°	36.148°	30.123°
**Length of Mean Vector (r)**	0.925	0.842	0.595	0.736	0.982	0.061	0.591	0.604
**Median**	24.555°	31.669°	33.062°	16.555°	15.986°	41.158°	35.756°	33.923°
**Concentration**	6.95	3.498	1.49	1.486	13.596	0.123	1.473	1.523
**Circular Variance**	0.037	0.079	0.202	0.132	0.009	0.469	0.204	0.198
**Circular Standard Deviation**	11.309°	16.782°	29.18°	22.423°	5.502°	67.668°	29.374°	28.789°
**Standard Error of Mean**	1.552°	1.505°	3.012°	9.684°	3.507°	65.876°	3.898°	5.975°
**95% Confidence Interval (-/+) for μ**	21.84°	28.55°	28.047°	353.029°	8.172°	263°	28.505°	18.409°
	27.925°	34.45°	39.856°	30.996°	21.922°	161.286°	43.79°	41.837°
**99% Confidence Interval (-/+) for μ**	20.884°	27.623°	26.192°	347.066°	6.013°	222.433°	26.105°	14.73°
	28.881°	35.376°	41.711°	36.959°	24.081°	201.852°	46.191°	45.516°
**Rayleigh Test (Z)**	45.352	87.272	36.144	4.335	4.819	0.094	21.667	9.106
**Rayleigh Test (p)**	< 1E-12	< 1E-12	< 1E-12	0.009	0.002	0.912	3.89E-10	4.85E-05
**Watson's U^2 Test (von Mises, U^2)**	0.172	1.923	0.488	*****	*****	0.296	1.313	0.594
**Watson's U^2 Test (p)**	< 0.01	< 0.005	< 0.005	*****	*****	< 0.005	< 0.005	< 0.005
**Circular-linear correlation (r)**	0.226	0.308	0.293	0.63	0.792	0.156	0.314	0.235
**Circular-linear correlation (P)**	0.077	1.09E-05	2.00E-04	0.123	0.23	0.587	0.003	0.298

***** indicates that a result could not be calculated.

Table S4 (cont.).

Experiment and side	Exp 256 Left			Exp 257 Right			Exp 257 Left		
**Bite point**	Front	WS	BS	Front	WS	BS	Front	WS	BS
**Number of Observations**	28	25	65	30	11	5	30	5	11
**Mean Vector (µ)**	60.965°	55.245°	71.266°	178.516°	134.265°	151.687°	111.789°	167.294°	165.804°
**Length of Mean Vector (r)**	0.23	0.889	0.304	0.52	0.909	0.85	0.143	0.985	0.894
**Median**	64.473°	56.915°	69.357°	6.83°	136.95°	145.953°	117.15°	165.329°	170.327°
**Concentration**	0.473	4.803	0.639	1.214	4.31	1.795	0.289	16.583	3.726
**Circular Variance**	0.385	0.055	0.348	0.24	0.045	0.075	0.428	0.007	0.053
**Circular Standard Deviation**	49.12°	13.893°	44.192°	32.743°	12.511°	16.337°	56.501°	4.974°	13.578°
**Standard Error of Mean**	16.424°	2.773°	8.059°	6.582°	4.364°	10.372°	25.729°	3.17°	4.734°
**95% Confidence Interval (-/+) for µ**	28.768°	49.809°	55.467°	165.613°	125.71°	131.355°	61.349°	161.08°	156.524°
	93.162°	60.681°	87.065°	191.419°	142.82°	172.02°	162.228°	173.509°	175.084°
**99% Confidence Interval (-/+) for µ**	18.655°	48.102°	50.504°	161.56°	123.023°	124.968°	45.505°	159.128°	153.61°
	103.275°	62.388°	92.028°	195.472°	145.507°	178.407°	178.072°	175.461°	177.998°
**Rayleigh Test (Z)**	1.48	19.761	6.018	8.124	9.09	3.612	0.613	4.852	8.787
**Rayleigh Test (p)**	0.229	5.60E-09	0.002	1.81E-04	< 1E-12	0.018	0.545	0.002	< 1E-12
**Watson's U^2 Test (von Mises, U^2)**	0.323	0.249	0.685	0.32	0.195	*****	0.046	*****	0.276
**Watson's U^2 Test (p)**	< 0.005	< 0.005	< 0.005	< 0.005	< 0.005	*****	0.25 > p > 0.15	*****	< 0.005
**Circular-linear correlation (r)**	0.205	0.236	0.34	0.66	0.678	0.572	0.776	0.468	0.483
**Circular-linear correlation (P)**	0.351	0.293	7.37E-04	3.35E-06	0.019	0.506	6.97E-09	0.641	0.15

***** indicates that a result could not be calculated.

Table S5. Descriptive circular statistics for *in vivo* bone strain orientations in the jugal of *Uromastyx*, sorted by food type.

**Experiment and side**	**Exp 220 Right**			**Exp 220 Left**			**Exp 256 Right**	
**Food type**	Greens	Mazuri	Transducer	Greens	Mazuri	Transducer	Greens	Mazuri
**Number of Observations**	221	129	39	15	41	11	415	128
**Mean Vector (µ)**	33.827°	31.072°	22.43°	10.495°	179.525°	179.373°	27.406°	41.721°
**Length of Mean Vector (r)**	0.77	0.72	0.918	0.46	0.825	0.41	0.246	0.068
**Median**	32.65°	28.857°	20.771°	179.393°	179.159°	15.461°	32.033°	42.64°
**Concentration**	2.54	2.138	6.417	0.905	3.198	0.697	0.507	0.135
**Circular Variance**	0.115	0.14	0.041	0.27	0.088	0.295	0.377	0.466
**Circular Standard Deviation**	20.712°	23.209°	11.814°	35.705°	17.792°	38.241°	48.009°	66.513°
**Standard Error of Mean**	1.378°	2.033°	1.89°	11.463°	2.755°	16.151°	3.987°	26.486°
**95% Confidence Interval (-/+) for µ**	31.126°	27.087°	18.725°	348.023°	174.124°	147.71°	19.59°	349.797°
	36.529°	35.056°	26.134°	32.967°	184.926°	211.036°	35.221°	93.644°
**99% Confidence Interval (-/+) for µ**	30.278°	25.835°	17.562°	340.964°	172.427°	137.764°	17.135°	333.487°
	37.377°	36.308°	27.298°	40.025°	186.623°	220.981°	37.676°	109.955°
**Rayleigh Test (Z)**	131.033	66.917	32.901	3.173	27.879	1.852	25.026	0.584
**Rayleigh Test (p)**	< 1E-12	< 1E-12	< 1E-12	0.039	3.62E-12	0.158	1.35E-11	0.558
**Watson's U^2 Test (von Mises, U^2)**	2.473	0.635	0.176	0.145	0.051	0.14	6.471	1.602
**Watson's U^2 Test (p)**	< 0.005	< 0.005	< 0.01	< 0.005	0.5 > p > 0.25	< 0.005	< 0.005	< 0.005
**Circular-linear correlation (r)**	0.331	0.19	0.342	0.593	0.398	0.672	0.191	0.18
**Circular-linear correlation (P)**	3.60E-11	0.011	0.015	0.012	0.002	0.02	2.88E-07	0.018

Table S5 (cont.).

Experiment and side	Exp 256 Left		Exp 257 Right		Exp 257 Left	
**Food type**	Greens	Mazuri	Greens	Mazuri	Greens	Mazuri
**Number of Observations**	421	208	275	64	275	64
**Mean Vector (μ)**	60.51°	160.019°	157.109°	112.807°	160.894°	163.883°
**Length of Mean Vector (r)**	0.372	0.252	0.585	0.612	0.509	0.41
**Median**	60.371°	154.669°	149.356°	106.376°	162.535°	172.031°
**Concentration**	0.803	0.52	1.45	1.56	1.177	0.898
**Circular Variance**	0.314	0.374	0.207	0.194	0.246	0.295
**Circular Standard Deviation**	40.263°	47.587°	29.651°	28.378°	33.312°	38.264°
**Standard Error of Mean**	2.554°	5.49°	1.875°	3.664°	2.233°	5.902°
**95% Confidence Interval (-/+) for μ**	55.503°	149.256°	153.433°	105.624°	156.517°	152.312°
	65.516°	170.781°	160.784°	119.991°	165.271°	175.454°
**99% Confidence Interval (-/+) for μ**	53.931°	145.875°	152.278°	103.368°	155.142°	148.677°
	67.089°	174.162°	161.939°	122.247°	166.645°	179.089°
**Rayleigh Test (Z)**	58.402	13.175	94.207	23.991	71.142	10.749
**Rayleigh Test (p)**	< 1E-12	1.90E-06	< 1E-12	3.81E-11	< 1E-12	2.15E-05
**Watson's U^2 Test (von Mises, U^2)**	5.533	2.304	2.256	0.298	0.752	0.732
**Watson's U^2 Test (p)**	< 0.005	< 0.005	< 0.005	< 0.005	< 0.005	< 0.005
**Circular-linear correlation (r)**	0.453	0.329	0.501	0.621	0.536	0.41
**Circular-linear correlation (P)**	< 1E-12	2.14E-10	< 1E-12	1.67E-11	< 1E-12	3.19E-05

Table S6. Descriptive circular statistics for *in vivo* bone strain orientations in the jugal of *Uromastyx*, sorted by feeding behavior.

**Experiment and side**	**Exp 220 Right**							
**Behavior**	Flick	Capture	Manipulation	Crush	Chew	Swallow	Lick	Tear
**Number of Observations**	4	4	128	5	175	11	46	4
**Mean Vector (µ)**	53.414°	52.609°	31.104°	22.069°	31.816°	40.633°	28.838°	26.211°
**Length of Mean Vector (r)**	0.981	0.995	0.751	0.95	0.776	0.73	0.695	0.956
**Median**	50.399°	51.488°	29.508°	20.433°	31.653°	35.476°	26.105°	30.056°
**Concentration**	10.699	37.38	2.369	5.009	2.599	1.643	1.977	4.658
**Circular Variance**	0.009	0.003	0.125	0.025	0.112	0.135	0.152	0.022
**Circular Standard Deviation**	5.571°	2.96°	21.689°	9.221°	20.4°	22.746°	24.426°	8.556°
**Standard Error of Mean**	4.421°	2.349°	1.898°	5.875°	1.525°	7.889°	3.603°	6.787°
**95% Confidence Interval (-/+) for µ**	44.747°	48.004°	27.382°	10.552°	28.826°	25.167°	21.776°	12.906°
	62.08°	57.215°	34.826°	33.586°	34.805°	56.099°	35.9°	39.516°
**99% Confidence Interval (-/+) for µ**	42.025°	46.558°	26.213°	6.934°	27.887°	20.308°	19.557°	8.727°
	64.802°	58.661°	35.995°	37.204°	35.744°	60.958°	38.119°	43.695°
**Rayleigh Test (Z)**	3.852	3.958	72.159	4.508	105.395	5.856	22.235	3.659
**Rayleigh Test (p)**	0.009	0.008	**< 1E-12**	0.004	< 1E-12	0.001	1.99E-10	0.014
**Watson's U² Test (von Mises, U²)**	*****	*****	0.996	*****	1.691	0.068	0.325	*****
**Watson's U² Test (p)**	*****	*****	< 0.005	*****	< 0.005	0.25 > p > 0.15	< 0.005	*****
**Circular-linear correlation (r)**	0.997	0.848	0.315	0.484	0.284	0.254	0.238	0.865
**Circular-linear correlation (P)**	0.057	0.405	4.06E-06	0.62	9.52E-07	0.596	0.088	0.38

***** indicates that a result could not be calculated.

Table S6 (cont.).

**Experiment and side**	**Exp 220 Left**					
**Behavior**	Flick	Capture	Manipulation	Chew	Swallow	Lick
**Number of Observations**	4	3	17	10	4	19
**Mean Vector (µ)**	171.345°	165.614°	13.579°	5.193°	169.99°	3.116°
**Length of Mean Vector (r)**	0.99	1	0.648	0.564	0.988	0.791
**Median**	172.091°	165.835°	17.549°	92.87°	170.041°	0.187°
**Concentration**	19.964	311.081	1.721	1.223	17.253	2.761
**Circular Variance**	0.005	2.14E-04	0.176	0.218	0.006	0.104
**Circular Standard Deviation**	4.061°	0.839°	26.697°	30.672°	4.371°	19.597°
**Standard Error of Mean**	3.222°	0.938°	6.58°	10.909°	3.469°	4.446°
**95% Confidence Interval (-/+) for µ**	165.029°	163.775°	0.679°	343.806°	163.19°	354.4°
	177.661°	167.453°	26.479°	26.58°	176.79°	11.832°
**99% Confidence Interval (-/+) for µ**	163.045°	163.198°	356.627°	337.088°	161.054°	351.662°
	179.646°	168.031°	30.531°	33.298°	178.926°	14.57°
**Rayleigh Test (Z)**	3.92	2.997	7.133	3.178	3.908	11.899
**Rayleigh Test (p)**	0.008	0.034	3.90E-04	0.037	0.008	8.90E-07
**Watson's U^2 Test (von Mises, U^2)**	*****	*****	0.207	0.038	*****	0.265
**Watson's U^2 Test (p)**	*****	*****	< 0.005	0.5 > p > 0.25	*****	< 0.005
**Circular-linear correlation (r)**	0.999	1	0.356	0.92	0.913	0.291
**Circular-linear correlation (P)**	0.027	1	0.169	1.66E-04	0.301	0.257

***** indicates that a result could not be calculated.

Table S6 (cont.).

**Experiment and side**	**Exp 256 Right**						
**Behavior**	Flick	Capture	Manipulation	Crush	Chew	Swallow	Lick
**Number of Observations**	9	48	136	23	214	91	9
**Mean Vector (µ)**	131.394°	157.209°	30.556°	35.452°	33.888°	125.265°	126.08°
**Length of Mean Vector (r)**	0.782	0.119	0.634	0.813	0.412	0.725	0.996
**Median**	130.223°	152.891°	32.871°	37.064°	33.928°	124.955°	126.226°
**Concentration**	1.842	0.239	1.654	3.025	0.904	2.168	85.147
**Circular Variance**	0.109	0.441	0.183	0.094	0.294	0.138	0.002
**Circular Standard Deviation**	20.114°	59.125°	27.368°	18.453°	38.146°	22.997°	2.591°
**Standard Error of Mean**	7.959°	24.51°	2.399°	3.81°	3.208°	2.396°	1.037°
**95% Confidence Interval (-/+) for µ**	115.79°	109.161°	25.852°	27.983°	27.598°	120.568°	124.047°
	146.997°	205.258°	35.26°	42.921°	40.177°	129.962°	128.113°
**99% Confidence Interval (-/+) for µ**	110.889°	94.068°	24.375°	25.637°	25.622°	119.092°	123.408°
	151.898°	220.351°	36.737°	45.268°	42.153°	131.437°	128.751°
**Rayleigh Test (Z)**	5.497	0.678	54.599	15.19	36.34	47.772	8.927
**Rayleigh Test (p)**	0.002	0.51	< 1E-12	1.05E-07	< 1E-12	< 1E-12	< 1E-12
**Watson's U^2 Test (von Mises, U^2)**	*****	0.415	3.504	0.631	3.906	1.846	*****
**Watson's U^2 Test (p)**	*****	< 0.005	< 0.005	< 0.005	< 0.005	< 0.005	*****
**Circular-linear correlation (r)**	0.551	0.381	0.303	0.484	0.224	0.448	0.491
**Circular-linear correlation (P)**	0.152	0.001	4.79E-06	0.008	2.39E-05	1.63E-08	0.229

***** indicates that a result could not be calculated.

Table S6 (cont.).

**Experiment and side**	**Exp 256 Left**						
**Behavior**	Flick	Capture	Manipulation	Crush	Chew	Swallow	Lick
**Number of Observations**	13	56	143	36	230	120	18
**Mean Vector (μ)**	157.66°	167.845°	60.217°	59.745°	60.54°	155.251°	152.424°
**Length of Mean Vector (r)**	0.394	0.11	0.606	0.735	0.403	0.587	0.993
**Median**	154.058°	171.176°	60.549°	60.273°	60.6°	154.282°	152.741°
**Concentration**	0.678	0.222	1.534	2.242	0.88	1.456	72.996
**Circular Variance**	0.303	0.445	0.197	0.133	0.299	0.207	0.003
**Circular Standard Deviation**	39.099°	60.14°	28.668°	22.496°	38.637°	29.583°	3.365°
**Standard Error of Mean**	15.377°	24.441°	2.484°	3.72°	3.174°	2.829°	0.793°
**95% Confidence Interval (-/+) for μ**	127.514°	119.931°	55.347°	52.452°	54.319°	149.704°	150.869°
	187.806°	215.759°	65.088°	67.038°	66.762°	160.798°	153.979°
**99% Confidence Interval (-/+) for μ**	118.045°	104.88°	53.817°	50.161°	52.364°	147.962°	150.381°
	197.275°	230.81°	66.618°	69.329°	68.717°	162.54°	154.467°
**Rayleigh Test (Z)**	2.018	0.683	52.532	19.432	37.305	41.312	17.753
**Rayleigh Test (p)**	0.133	0.505	< 1E-12	2.63E-09	< 1E-12	< 1E-12	5.03E-08
**Watson's U² Test (von Mises, U²)**	0.256	0.583	1.976	0.394	3.013	2.215	0.316
**Watson's U² Test (p)**	< 0.005	< 0.005	< 0.005	< 0.005	< 0.005	< 0.005	< 0.005
**Circular-linear correlation (r)**	0.356	0.282	0.322	0.223	0.444	0.26	0.708
**Circular-linear correlation (P)**	0.279	0.015	4.66E-07	0.193	< 1E-12	3.61E-04	2.60E-04

***** indicates that a result could not be calculated.

Table S6 (cont.).

**Experiment and side**	**Exp 257 Right**						
**Behavior**	Flick	Capture	Manipulation	Crush	Chew	Swallow	Lick
**Number of Observations**	10	51	103	4	108	34	26
**Mean Vector (µ)**	172.37°	161.264°	159.361°	137.73°	152.589°	103.141°	107.996°
**Length of Mean Vector (r)**	0.418	0.483	0.706	0.996	0.613	0.666	0.637
**Median**	82.71°	150.467°	150.672°	137.04°	147.877°	104.251°	104.275°
**Concentration**	0.703	1.102	2.042	45.349	1.561	1.812	1.671
**Circular Variance**	0.291	0.258	0.147	0.002	0.194	0.167	0.181
**Circular Standard Deviation**	37.82°	34.538°	23.917°	2.687°	28.362°	25.842°	27.193°
**Standard Error of Mean**	16.701°	5.496°	2.351°	2.132°	2.819°	4.474°	5.444°
**95% Confidence Interval (-/+) for µ**	139.629°	150.49°	154.751°	133.551°	147.063°	94.371°	97.324°
	205.11°	172.037°	163.97°	141.909°	158.114°	111.911°	118.667°
**99% Confidence Interval (-/+) for µ**	129.345°	147.105°	153.304°	132.238°	145.328°	91.616°	93.972°
	215.395°	175.422°	165.418°	143.221°	159.85°	114.666°	122.019°
**Rayleigh Test (Z)**	1.75	11.921	51.302	3.965	40.529	15.069	10.56
**Rayleigh Test (p)**	0.176	6.65E-06	< 1E-12	0.008	< 1E-12	7.24E-08	8.30E-06
**Watson's U^2 Test (von Mises, U^2)**	0.16	0.434	1.316	*****	1.045	0.185	0.403
**Watson's U^2 Test (p)**	< 0.005	< 0.005	< 0.005	*****	< 0.005	< 0.005	< 0.005
**Circular-linear correlation (r)**	0.705	0.634	0.365	0.748	0.457	0.511	0.941
**Circular-linear correlation (P)**	0.022	1.31E-09	1.56E-06	0.531	2.23E-10	2.55E-04	< 1E-12

***** indicates that a result could not be calculated.

Table S6 (cont.).

**Experiment and side**	**Exp 257 Left**						
**Behavior**	Flick	Capture	Manipulation	Crush	Chew	Swallow	Lick
**Number of Observations**	10	51	103	4	108	34	26
**Mean Vector (µ)**	99.344°	125.68°	167.768°	170.866°	160.342°	104.288°	178.06°
**Length of Mean Vector (r)**	0.324	0.435	0.857	0.997	0.545	0.088	0.549
**Median**	115.291°	120.043°	165.819°	170.075°	161.472°	106.751°	178.258°
**Concentration**	0.395	0.964	3.803	62.448	1.302	0.176	1.315
**Circular Variance**	0.338	0.283	0.072	0.002	0.228	0.456	0.226
**Circular Standard Deviation**	42.982°	36.98°	15.927°	2.288°	31.571°	63.198°	31.396°
**Standard Error of Mean**	25.317°	6.196°	1.564°	1.815°	3.273°	39.514°	6.616°
**95% Confidence Interval (-/+) for µ**	49.713°	113.535°	164.702°	167.307°	153.926°	26.825°	165.09°
	148.976°	137.826°	170.833°	174.425°	166.759°	181.75°	191.029°
**99% Confidence Interval (-/+) for µ**	34.123°	109.719°	163.739°	166.189°	151.911°	2.493°	161.016°
	164.566°	141.641°	171.796°	175.543°	168.774°	206.083°	195.103°
**Rayleigh Test (Z)**	1.053	9.637	75.614	3.975	32.06	0.262	7.823
**Rayleigh Test (p)**	0.358	6.53E-05	< 1E-12	0.007	< 1E-12	0.772	2.36E-04
**Watson's U^2 Test (von Mises, U^2)**	0.064	0.167	0.161	*****	0.303	0.168	0.531
**Watson's U^2 Test (p)**	0.1 > p > 0.05	< 0.005	< 0.01	*****	< 0.005	< 0.005	< 0.005
**Circular-linear correlation (r)**	0.619	0.585	0.416	1	0.523	0.257	0.304
**Circular-linear correlation (P)**	0.059	3.62E-08	2.52E-08	0.006	< 1E-12	0.128	0.119

***** indicates that a result could not be calculated.

Table S7. Results of Mardia-Watson-Wheeler tests to determine whether ε1 angle distributions are identical within gage sites, sorted by bite location.

**Experiment and side**	**W**	**p**
Exp 220 Right		
Front E1 angle & WS E1 angle	17.431	1.64E-04
Front E1 angle & BS E1 angle	26.519	1.74E-06
WS E1 angle & BS E1 angle	25.527	2.86E-06
Exp 220 Left		
WS E1 angle & BS E1 angle	*****	*****
Exp 256 Right		
Front E1 angle & WS E1 angle	7.408	0.025
Front E1 angle & BS E1 angle	12.697	0.002
WS E1 angle & BS E1 angle	6.851	0.033
Exp 256 Left		
Front E1 angle & WS E1 angle	20.159	4.19E-05
Front E1 angle & BS E1 angle	1.895	0.388
WS E1 angle & BS E1 angle	34.748	2.85E-08
Exp 257 Right		
Front E1 angle & WS E1 angle	14.208	8.22E-04
Front E1 angle & BS E1 angle	*****	*****
WS E1 angle & BS E1 angle	*****	*****
Exp 257 Left		
Front E1 angle & WS E1 angle	*****	*****
Front E1 angle & BS E1 angle	16.45	2.68E-04
WS E1 angle & BS E1 angle	*****	*****

***** indicates that a result could not be calculated.

Table S8. Results of Mardia-Watson-Wheeler tests to determine whether ε1 angle distributions are identical within gage sites, sorted by food type.

Experiment and side	W	p
Exp 220 Right		
Greens E1 angle & Mazuri E1 angle	29.314	4.31E-07
Greens E1 angle & Transducer E1 angle	40.127	1.93E-09
Mazuri E1 angle & Transducer E1 angle	11.366	0.003
Exp 220 Left		
Greens E1 angle & Mazuri E1 angle	3.882	0.144
Greens E1 angle & Transducer E1 angle	1.258	0.533
Mazuri E1 angle & Transducer E1 angle	17.01	2.02E-04
Exp 256 Right		
Greens E1 angle & Mazuri E1 angle	48.239	3.35E-11
Exp 256 Left		
Greens E1 angle & Mazuri E1 angle	60.079	< 1E-12
Exp 257 Right		
Greens E1 angle & Mazuri E1 angle	103.863	< 1E-12
Exp 257 Left		
Greens E1 angle & Mazuri E1 angle	16.444	2.69E-04

Table S9. Results of Mardia-Watson-Wheeler tests to determine whether ε1 angle distributions are identical within gage sites, sorted by feeding behavior.

Experiment and side	W	p
Exp 220 Right		
Flick E1 angle & Capture E1 angle	*****	*****
Flick E1 angle & Manip. E1 angle	*****	*****
Flick E1 angle & Crush E1 angle	*****	*****
Flick E1 angle & Chew E1 angle	*****	*****
Flick E1 angle & Swallow E1 angle	*****	*****
Flick E1 angle & Lick E1 angle	*****	*****
Flick E1 angle & Tear E1 angle	*****	*****
Capture E1 angle & Manip. E1 angle	*****	*****
Capture E1 angle & Crush E1 angle	*****	*****
Capture E1 angle & Chew E1 angle	*****	*****
Capture E1 angle & Swallow E1 angle	*****	*****
Capture E1 angle & Lick E1 angle	*****	*****
Capture E1 angle & Tear E1 angle	*****	*****
Manip. E1 angle & Crush E1 angle	*****	*****
Manip. E1 angle & Chew E1 angle	9.152	0.01
Manip. E1 angle & Swallow E1 angle	5.904	0.052
Manip. E1 angle & Lick E1 angle	4.8	0.091
Manip. E1 angle & Tear E1 angle	*****	*****
Crush E1 angle & Chew E1 angle	*****	*****
Crush E1 angle & Swallow E1 angle	*****	*****
Crush E1 angle & Lick E1 angle	*****	*****
Crush E1 angle & Tear E1 angle	*****	*****
Chew E1 angle & Swallow E1 angle	4.244	0.12
Chew E1 angle & Lick E1 angle	14.349	7.66E-04
Chew E1 angle & Tear E1 angle	*****	*****
Swallow E1 angle & Lick E1 angle	5.67	0.059
Swallow E1 angle & Tear E1 angle	*****	*****
Lick E1 angle & Tear E1 angle	*****	*****
Exp 220 Left		
Flick E1 angle & Capture E1 angle	*****	*****
Flick E1 angle & Manip. E1 angle	*****	*****
Flick E1 angle & Chew E1 angle	*****	*****
Flick E1 angle & Swallow E1 angle	*****	*****
Flick E1 angle & Lick E1 angle	*****	*****
Capture E1 angle & Manip. E1 angle	*****	*****
Capture E1 angle & Chew E1 angle	*****	*****
Capture E1 angle & Swallow E1 angle	*****	*****
Capture E1 angle & Lick E1 angle	*****	*****

Table S9 (cont.).

Experiment and side	W	p
Exp 220 Left		
Manip.E1 andlg & Chew E1 angle	6.312	0.043
Manip. E1 angle & Swallow E1 angle	*****	*****
Manip. E1 angle & Lick E1 angle	19.123	7.04E-05
Chew E1 angle & Swallow E1 angle	*****	*****
Chew E1 angle & Lick E1 angle	6.705	0.035
Swallow E1 angle & Lick E1 angle	*****	*****
Exp 256 Right		
Flick E1 angle & Capture E1 angle	*****	*****
Flick E1 angle & Manip. E1 angle	*****	*****
Flick E1 angle & Crush E1 angle	*****	*****
Flick E1 angle & Chew E1 angle	*****	*****
Flick E1 angle & Swallow E1 angle	*****	*****
Flick E1 angle & Lick E1 angle	*****	*****
Capture E1 angle & Manip. E1 angle	32.137	1.05E-07
Capture E1 angle & Crush E1 angle	19.108	7.09E-05
Capture E1 angle & Chew E1 angle	16.629	2.45E-04
Capture E1 angle & Swallow E1 angle	16.796	2.25E-04
Capture E1 angle & Lick E1 angle	*****	*****
Manip. E1 angle & Crush E1 angle	15.282	4.80E-04
Manip. E1 angle & Chew E1 angle	22.608	1.23E-05
Manip. E1 angle & Swallow E1 angle	115.881	< 1E-12
Manip. E1 angle & Lick E1 angle	*****	*****
Crush E1 angle & Chew E1 angle	7.191	0.027
Crush E1 angle & Swallow E1 angle	35.896	1.60E-08
Crush E1 angle & Lick E1 angle	*****	*****
Chew E1 angle & Swallow E1 angle	81.005	< 1E-12
Chew E1 angle & Lick E1 angle	*****	*****
Swallow E1 angle & Lick E1 angle	*****	*****
Exp 256 Left		
Flick E1 angle & Capture E1 angle	1.401	0.496
Flick E1 angle & Manip. E1 angle	11.843	0.003
Flick E1 angle & Crush E1 angle	10.349	0.006
Flick E1 angle & Chew E1 angle	9.432	0.009
Flick E1 angle & Swallow E1 angle	1.135	0.567
Flick E1 angle & Lick E1 angle	12.277	0.002
Capture E1 angle & Manip. E1 angle	16.284	2.91E-04
Capture E1 angle & Crush E1 angle	11.449	0.003
Capture E1 angle & Chew E1 angle	10.527	0.005
Capture E1 angle & Swallow E1 angle	11.16	0.004
Capture E1 angle & Lick E1 angle	24.289	5.32E-06
Manip. E1 angle & Crush E1 angle	0.183	0.913

Table S9 (cont.).

Experiment and side	W	p
Exp 256 Left		
Manip. E1 angle & Chew E1 angle	2.259	0.323
Manip. E1 angle & Swallow E1 angle	83.745	< 1E-12
Manip. E1 angle & Lick E1 angle	37.653	6.67E-09
Crush E1 angle & Chew E1 angle	1.482	0.477
Crush E1 angle & Swallow E1 angle	45.889	1.08E-10
Crush E1 angle & Lick E1 angle	36.973	9.36E-09
Chew E1 angle & Swallow E1 angle	76.419	< 1E-12
Chew E1 angle & Lick E1 angle	36.954	9.45E-09
Swallow E1 angle & Lick E1 angle	17.655	1.47E-04
Exp 257 Right		
Flick E1 angle & Capture E1 angle	1.935	0.38
Flick E1 angle & Manip. E1 angle	15.056	5.38E-04
Flick E1 angle & Crush E1 angle	*****	*****
Flick E1 angle & Chew E1 angle	9.388	0.009
Flick E1 angle & Swallow E1 angle	20.556	3.44E-05
Flick E1 angle & Lick E1 angle	16.106	3.18E-04
Capture E1 angle & Manip. E1 angle	12.919	0.002
Capture E1 angle & Crush E1 angle	*****	*****
Capture E1 angle & Chew E1 angle	6.261	0.044
Capture E1 angle & Swallow E1 angle	49.974	1.41E-11
Capture E1 angle & Lick E1 angle	35.917	1.59E-08
Manip. E1 angle & Crush E1 angle	*****	*****
Manip. E1 angle & Chew E1 angle	2.309	0.315
Manip. E1 angle & Swallow E1 angle	64.764	< 1E-12
Manip. E1 angle & Lick E1 angle	54.102	1.79E-12
Crush E1 angle & Chew E1 angle	*****	*****
Crush E1 angle & Swallow E1 angle	*****	*****
Crush E1 angle & Lick E1 angle	*****	*****
Chew E1 angle & Swallow E1 angle	55.045	1.11E-12
Chew E1 angle & Lick E1 angle	45.507	1.31E-10
Swallow E1 angle & Lick E1 angle	3.159	0.206
Exp 257 Left		
Flick E1 angle & Capture E1 angle	2.069	0.355
Flick E1 angle & Manip. E1 angle	14.3	7.85E-04
Flick E1 angle & Crush E1 angle	*****	*****
Flick E1 angle & Chew E1 angle	8.376	0.015
Flick E1 angle & Swallow E1 angle	0.177	0.915
Flick E1 angle & Lick E1 angle	8.048	0.018
Capture E1 angle & Manip. E1 angle	41.564	9.43E-10
Capture E1 angle & Crush E1 angle	*****	*****
Capture E1 angle & Chew E1 angle	16.571	2.52E-04

Table S9 (cont.).

Experiment and side	W	p
Exp 257 Left		
Capture E1 angle & Swallow E1 angle	9.991	0.007
Capture E1 angle & Lick E1 angle	19.452	5.97E-05
Manip. E1 angle & Crush E1 angle	*****	*****
Manip. E1 angle & Chew E1 angle	14.525	7.01E-04
Manip. E1 angle & Swallow E1 angle	32.287	9.75E-08
Manip. E1 angle & Lick E1 angle	21.335	2.33E-05
Crush E1 angle & Chew E1 angle	*****	*****
Crush E1 angle & Swallow E1 angle	*****	*****
Crush E1 angle & Lick E1 angle	*****	*****
Chew E1 angle & Swallow E1 angle	23.217	9.09E-06
Chew E1 angle & Lick E1 angle	27.269	1.20E-06
Swallow E1 angle & Lick E1 angle	12.665	0.002

***** indicates that a result could not be calculated.

Table S10. ANOVA testing for differences in ε1 and ε2 and shear strain magnitudes and principal strain ratios due to food type and behavior. All individuals were included in right gage analyses; only *Uro1* and *Uro7* were included in left gage analyses. Behaviors analyzed include: captures (left only), manipulations, chews, swallows and licks.

E1

RIGHT GAGE				LEFT GAGE			
Source	*d.f.*	*F*	*P*	*Source*	*d.f.*	*F*	*P*
Food	1,1029	**31.9679**	**<0.0001**	Food	1,875.1	0.8029	0.3705
Behavior	3,1030	**27.6119**	**<0.0001**	Behavior	4,875	**34.8462**	**<0.0001**
Food*Behavior	3,1030	2.4535	0.0619	Food*Behavior	4,875	**3.4904**	**0.0077**

E2

RIGHT GAGE				LEFT GAGE			
Source	*d.f.*	*F*	*P*	*Source*	*d.f.*	*F*	*P*
Food	1,1025	2.1888	0.1339	Food	1,876.1	0.8057	0.3696
Behavior	3,1026	**87.5332**	**<0.0001**	Behavior	4,876.1	**56.7883**	**<0.0001**
Food*Behavior	3,1026	**2.7081**	**0.0441**	Food*Behavior	4,876	**9.6662**	**<0.0001**

Shear strain

RIGHT GAGE				LEFT GAGE			
Source	*d.f.*	*F*	*P*	*Source*	*d.f.*	*F*	*P*
Food	1,1032	**19.8208**	**<0.0001**	Food	1,879	0.4188	0.5177
Behavior	3,1033	**72.9793**	**<0.0001**	Behavior	4,879	**59.4123**	**<0.0001**
Food*Behavior	3,1032	1.9731	0.1163	Food*Behavior	4,879	**8.2731**	**<0.0001**

|ε1 / ε2|

RIGHT GAGE				LEFT GAGE			
Source	*d.f.*	*F*	*P*	*Source*	*d.f.*	*F*	*P*
Food	1,1032	0.1069	0.7438	Food	1,879.7	**6.2823**	**0.0124**
Behavior	3,1033	**82.1776**	**<0.0001**	Behavior	4,879.4	**6.5309**	**<0.0001**
Food*Behavior	3,1032	**2.9200**	**0.0331**	Food*Behavior	4,879.1	**2.9960**	**0.0180**

Table S11. ANOVA testing for differences in ε1 and ε2 and shear strain magnitudes due to bite location.

E1

RIGHT GAGE				LEFT GAGE			
Source	*d.f.*	*F*	*P*	*Source*	*d.f.*	*F*	*P*
Side	2,389.1	**25.6374**	**<0.0001**	Side	2,168.1	**9.4276**	**<0.0001**

E2

RIGHT GAGE				LEFT GAGE			
Source	*d.f.*	*F*	*P*	*Source*	*d.f.*	*F*	*P*
Side	2,395	**22.2243**	**<0.0001**	Side	2,170.3	**11.0651**	**<0.0001**

Shear strain

RIGHT GAGE				LEFT GAGE			
Source	*d.f.*	*F*	*P*	*Source*	*d.f.*	*F*	*P*
Side	2,400.4	**28.6351**	**<0.0001**	Side	2,170.3	**10.9370**	**<0.0001**

|ε1 / ε2|

RIGHT GAGE				LEFT GAGE			
Source	*d.f.*	*F*	*P*	*Source*	*d.f.*	*F*	*P*
Side	2,400	**5.5684**	**0.0041**	Side	2,166.2	**7.0780**	**0.0011**

Table S12. Two-way ANOVA testing for differences in ε1 orientation due to food type and behavior. All individuals were included in right gage analyses; *Uro1* and *Uro7* were included in left gage analyses. As individual variation could not be included as a random effect, individuals were tested separately.

***Uro5* (Experiment 220)**

RIGHT GAGE			
Source	*d.f.*	*F*	*P*
Food	1	1.4018	0.2373
Behavior	3	1.7444	0.1579
Food*Behavior	3	1.7618	0.1544

***Uro1* (Experiment 256)**

RIGHT GAGE				LEFT GAGE			
Source	*d.f.*	X^2	*P*	*Source*	*d.f.*	X^2	*P*
Food	2	**33.4681**	**<0.0001**	Food	2	**33.4681**	**<0.0001**
Behavior	6	**265.8169**	**<0.0001**	Behavior	6	**265.8169**	**<0.0001**
Food*Behavior	3	-22.7757	1	Food*Behavior	3	-22.7757	1

***Uro7* (Experiment 257)**

RIGHT GAGE				LEFT GAGE			
Source	*d.f.*	X^2	*P*	*Source*	*d.f.*	X^2	*P*
Food	2	**84.7385**	**<0.0001**	Food	2	3.4870	0.1749
Behavior	6	**132.4011**	**<0.0001**	Behavior	8	**115.6787**	**<0.0001**
Food*Behavior	3	-56.1329	1	Food*Behavior	4	4.7968	0.8910

Table S13. ANOVA testing for differences in ε1 orientation due to bite location. All individuals were included in right gage analyses; *Uro1* and *Uro7* were included in left gage analyses. As individual variation could not be included as a random effect, individuals were tested separately.

***Uro5* (Experiment 220)**

RIGHT GAGE			
Source	*d.f.*	*F*	*P*
Food	2	**3.5008**	**0.0315**

***Uro1* (Experiment 256)**

RIGHT GAGE				LEFT GAGE			
Source	*d.f.*	*F*	*P*	*Source*	*d.f.*	*F*	*P*
Food	2	0.3009	0.7407	Food	2	1.9170	0.1517

***Uro7* (Experiment 257)**

RIGHT GAGE				LEFT GAGE			
Source	*d.f.*	*F*	*P*	*Source*	*d.f.*	*F*	*P*
Food	2	**12.3231**	**<0.0001**	Food	2	**5.7552**	**0.0061**
